# Metformin and Its Sulfenamide Prodrugs Inhibit Human Cholinesterase Activity

**DOI:** 10.1155/2017/7303096

**Published:** 2017-07-09

**Authors:** Magdalena Markowicz-Piasecka, Joanna Sikora, Łukasz Mateusiak, Elżbieta Mikiciuk-Olasik, Kristiina M. Huttunen

**Affiliations:** ^1^Laboratory of Bioanalysis, Department of Pharmaceutical Chemistry, Drug Analysis and Radiopharmacy, Medical University of Lodz, ul. Muszyńskiego 1, 90-151 Lodz, Poland; ^2^Students Research Group, Laboratory of Bioanalysis, Department of Pharmaceutical Chemistry, Drug Analysis and Radiopharmacy, Medical University of Lodz, ul. Muszyńskiego 1, 90-151 Lodz, Poland; ^3^Department of Pharmaceutical Chemistry, Drug Analysis and Radiopharmacy, Medical University of Lodz, ul. Muszyńskiego 1, 90-151 Lodz, Poland; ^4^School of Pharmacy, Faculty of Health Sciences, University of Eastern Finland, Yliopistonranta 1C, POB 1627 70211 Kuopio, Finland

## Abstract

The results of epidemiological and pathophysiological studies suggest that type 2 diabetes mellitus (T2DM) may predispose to Alzheimer's disease (AD). The two conditions present similar glucose levels, insulin resistance, and biochemical etiologies such as inflammation and oxidative stress. The diabetic state also contributes to increased acetylcholinesterase (AChE) activity, which is one of the factors leading to neurodegeneration in AD. The aim of this study was to assess in vitro the effects of metformin, phenformin, and metformin sulfenamide prodrugs on the activity of human AChE and butyrylcholinesterase (BuChE) and establish the type of inhibition. Metformin inhibited 50% of the AChE activity at micromolar concentrations (2.35 *μ*mol/mL, mixed type of inhibition) and seemed to be selective towards AChE since it presented low anti-BuChE activity. The tested metformin prodrugs inhibited cholinesterases (ChE) at nanomolar range and thus were more active than metformin or phenformin. The cyclohexyl sulfenamide prodrug demonstrated the highest activity towards both AChE (IC_50_ = 890 nmol/mL, noncompetitive inhibition) and BuChE (IC_50_ = 28 nmol/mL, mixed type inhibition), while the octyl sulfenamide prodrug did not present anti-AChE activity, but exhibited mixed inhibition towards BuChE (IC_50_ = 184 nmol/mL). Therefore, these two bulkier prodrugs were concluded to be the most selective compounds for BuChE over AChE. In conclusion, it was demonstrated that biguanides present a novel class of inhibitors for AChE and BuChE and encourages further studies of these compounds for developing both selective and nonselective inhibitors of ChEs in the future.

## 1. Introduction

Type 2 diabetes mellitus (T2DM) is a complex, chronic, and progressive metabolic disease characterized by relative insulin deficiency, insulin resistance (primarily in fat, liver, and muscle cells), and high glucose levels in blood [1, 2]. Importantly, the disease can lead to severe impairments in almost all vital organs, including the brain.

A review of recent papers and epidemiological data shows an increased risk of developing Alzheimer's disease (AD), a common neurodegenerative disease characterized by progressive memory shortfall and neuronal loss, in people with T2DM [3–6]. The pathological characteristics of AD include extracellular amyloid plaques consisting of aggregated amyloid *β* protein (A*β*), intracellular neurofibrillary tangles (NFTs) comprising hyperphosphorylated tau protein, and neuronal loss [7]. Both acetylcholinesterase (AChE) and decreased acetylcholine (Ach) levels may play a role in the occurrence of AD, as it has been reported that abnormal AChE expression in the AD brain occurs in association with amyloid plaques and NFTs [8, 9]. A*β* peptides influence AChE levels, and as a consequence, A*β* may be responsible for the increased AChE protein levels around plaques. However, as García-Ayllón et al. have highlighted, the increase in AChE associated with NFTs has not yet been sufficiently explored [10].

Several competing hypotheses have been proposed in order to explain the cause of AD. The oldest, on which currently available anti-AD therapeutics are based, is the cholinergic hypothesis, which postulates that reduced synthesis of acetylcholine (ACh) is a factor in AD development. As the inhibition of AChE causes an increase in the concentration of ACh in cholinergic synapses, new and potent AChE inhibitors may be helpful in the treatment of AD [11].

AChE is a key enzyme in the cholinergic nervous system, and its levels are consistently decreased in the brain during AD development [12]. It has been well documented that the distribution of AChE molecular forms is particularly affected in the AD brain, but the pathological significance of these changes with regard to AChE species remains unknown. Another important issue regarding AChE in AD is that not all molecular forms of AChE are equally affected. It has been found that the proportion of G4 (tetramer) forms in AD brains is particularly depleted whereas the minor G1 (monomers) species are mostly preserved or even slightly increased [10]. It has been speculated that AChE plays a role in phases of cell development, such as neuronal differentiation, regulation of cell growth, or cell adhesion, which occur independently of its catalytic activity; a more detailed description of the role of AChE in AD pathogenesis is given by García-Ayllón et al. [10]. However, further studies are needed to elucidate the additional, noncatalytic functions of AChE, their association with different AChE variants, and their role in AD.

The mechanisms through which T2DM may predispose a patient to AD are not fully understood but may involve several factors including glucose levels, biochemical etiologies such as inflammation, and oxidative stress [13–15]. Several authors have reported that the relationship between diabetes and cognitive impairment may be associated with lowered insulin levels and its resistance. For instance, it has been established that insulin promotes synapse formation, neuronal stem cell activation, general cell growth, and neuroprotection [16]. Therefore, the disruption of insulin levels, insulin signalling, or insulin resistance in the brain can lead to the dysfunction and degeneration of neurons [17]. In addition, postmortem studies have found reduced neocortical levels of insulin and binding to insulin receptors in the brains of AD patients [18]. Deficiencies or impairments in insulin signalling may also intensify neurodegeneration by promoting the phosphorylation of tau [19]. Furthermore, insulin resistance has also been shown to promote A*β* accumulation and the progression of neurodegeneration in AD [20].

Some authors have also indicated a correlation between butyrylcholinesterase (BuChE) and insulin sensitivity [21], which implies that BuChE could have a crucial role in diabetes associated with insulin resistance [22]. The connection between BuChE activity and lipid and lipoprotein levels, stroke, preeclampsia, systemic lupus erythematosus, and cardiovascular disease has also been studied [23]. Moreover, BuChE protein levels were found to be elevated in the case of AD patients [24, 25] and they were also found to attenuate amyloid fibril formation [26].

Metformin is the most frequently used drug for the treatment of T2DM and is characterized by multidirectional biological activity: apart from hypoglycaemic activity, it exerts beneficial effects on mortality rate in diabetic patients, improves serum lipid profile, positively influences the process of haemostasis, which is often abnormal in diabetic patients, and stimulates the expression of genes responsible for cellular antioxidant defense mechanisms [27].

Recently, several papers have examined the use of metformin in the treatment of neurodegenerative diseases such as AD [28], amnestic mild cognitive impairment [29], and Parkinson's disease [30]. Curiously, other papers confirm a link between chronic administration of metformin and accumulation of *β*-amyloid aggregates [31–33]. For instance, Chen et al. report that metformin significantly increased the levels of extracellular and intracellular A*β* species, and that metformin magnified the total BACE1 (*β*-amyloid-converting enzyme 1) enzymatic activity twofold [34]. In contrast, Hettich et al. [35] claim that metformin markedly decreased BACE1 protein expression and activity in vitro and in vivo, thereby reducing the amount of BACE1 cleavage products and the production of A*β* [35]. However, even less is known regarding the effects of metformin on AChE activity. One in vivo study found that metformin at a dose of 100 mg/kg ameliorates scopolamine-induced memory impairments; however, no significant effect was observed on the scopolamine-induced increase in AChE activity [36].

**Figure 1:**
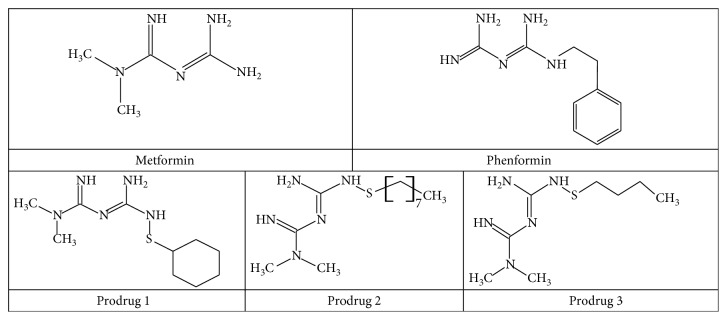
Chemical structure of biguanide derivatives: metformin, phenformin, and prodrugs 1–3.

Therefore, taking into consideration the multidirectional activity of metformin, the aim of the present study was to assess in vitro the effects of metformin, phenformin, and three selected sulfenamide metformin prodrugs (Figure 1) on the activity of human AChE and BuChE and to establish the type of inhibition. The findings will provide a greater insight into the more rational design of cholinesterase (ChE) inhibitors with a biguanide skeletal structure.

## 2. Materials and Methods

### 2.1. Materials

The design and synthesis of selected prodrugs 1–3 (Figure 1) was carried out at the University of Eastern Finland and reported elsewhere [37, 38].

We decided to choose 3 sulfenamide metformin prodrugs differing in their structure (length of alkyl chain or presence of cyclohexyl moiety), as well as physicochemical properties (distribution coefficients in octanol/water, bioconversion rate [37, 38]). The choice of prodrugs was made to find associations between the anticholinesterase activity and the structure of compounds. Not without significance were also previously conducted toxicity and biocompatibility studies (unpublished data). Studies concerning hemocompatibility of biguanides revealed that none of the tested prodrugs significantly affect the overall potential of clot formation and fibrinolysis (constant CL_AUC_), which indicate that the tested compounds can be regarded as biocompatible towards plasma haemostasis. The obtained PT, APTT, and fibrinogen concentrations demonstrate that the tested compounds do not interfere with the extrinsic and intrinsic coagulation pathways. The results of erythrotoxicity assays confirmed that the selected prodrugs are not toxic towards RBCs with exception of prodrug 2 at concentration of 1.5 *μ*mol/mL (13% hemolysis as compared to negative control).

The following reagents were used in this study: 0.9% NaCl (0.15 mol/L) (Chempur, Poland); 0.1 mol/L phosphate buffer pH = 7.0 and pH = 8.0 (disodium phosphate, monosodium phosphate (Baker, Poland)); a stock solution of 5,5′-dithiobisnitrobenzoic acid (DTNB; 0.01 mol/L (Sigma Aldrich, St. Louis, MO, USA)) prepared in phosphate buffer at pH = 7.0; a stock aqueous solution of acetylthiocholine iodide (21.67 mg/mL) (Sigma Aldrich); and a stock aqueous solution of butyrylthiocholine iodide (20.50 mg/mL) (Sigma Aldrich). All solutions were stored as small samples at a temperature of −30°C and before each experiment were restored at 37°C for 15 minutes.

### 2.2. Preparation of Biological Material

Blood samples were obtained from healthy donors from the Regional Blood Bank in Łódź, Poland (*Regionalne Centrum Krwiodawstwa i Krwiolecznictwa w Łodzi*). The blood was collected into vacuum tubes containing potassium versenate. Hemolysed human erythrocytes were used to determine AChE activity. Erythrocytes were separated from plasma by centrifugation (3000 ×g, 10 min, 20°C) with a Micro 22R centrifuge (Hettich Zentrifugen) and washed three times with 0.9% saline. Afterwards, red cells were hemolysed by freezing and stored at a temperature of −30°C; before each experiment, they were restored at 37°C for 15 minutes. Plasma for determination of BuChE activity was obtained by centrifuging the blood (3000 ×g, 10 min, 20°C).

The studies on biological material were approved by the Bioethics Committee of the Medical University of Lodz (RNN/109/16/KE).

### 2.3. Cholinesterase Inhibition

Prior to the study, probationary experiments were conducted to exclude potential interactions between sulfenamide prodrugs and reagents (DTNB, acetylthiocholine iodide, and butyrylthiocholine iodide). Spectrophotometric measurements of absorbance did not reveal any interactions between the reagents.

Acetylcholinesterase and butyrylcholinesterase activities were defined spectrophotometrically according to the Ellman method [39] with some modifications [40].

The experiments were performed on 96-well plates, and the absorbance was recorded at *λ* = 436 nm using a microplate reader (Synergy*™* H1 reader (Bio-Tek Instruments Inc., USA)). The diluted solution of hemolysed erythrocytes or diluted plasma was incubated for 10 minutes (37°C) with DTNB and tested compound at appropriate concentration, and the reaction was started by addition of substrate (acetylthiocholine iodide or butyrylthiocholine iodide). The absorbance was measured for five minutes, and the maximal velocity of the reaction was counted on the basis of changes in absorbance over time.

To validate the method, twelve control tests were conducted both for AChE and BuChE experiments. The coefficients of variability were counted (*W*_AChE_ = 0.055, *W*_BuChE_ = 0.072, resp.).

### 2.4. Kinetic Parameter Estimation

The experiments were conducted using decreasing concentrations (2-, 3-, 5-, 10-, and 20-fold) of substrate (acetylthiocholine iodide or butyrylthiocholine iodide). The absorbance was recorded at *λ* = 436 nm using a CECIL 2021 spectrophotometer (CECIL Cambridge, UK) with a thermostatic water flow (temperature 37°C).

### 2.5. Data Analysis

All values are expressed as mean ± SD. All experiments (in duplicates) were conducted three times on different biological samples.

The IC_50_ value, defined as the drug concentration that inhibits 50% of the activity of an enzyme, was determined by linear regression (*y* = *a*∗*x* + *b*). AChE SI (selectivity index) was calculated by using the following formula: SI = IC_50_ of BChE/IC_50_ of AChE.

The calculations of maximal velocity (*V*_max_) and the Michaelis constant (*K_m_*) were performed using linear regression (according to the Hanes-Woolf plot).

## 3. Results

### 3.1. Cholinesterase Activity

**Figure 2:**
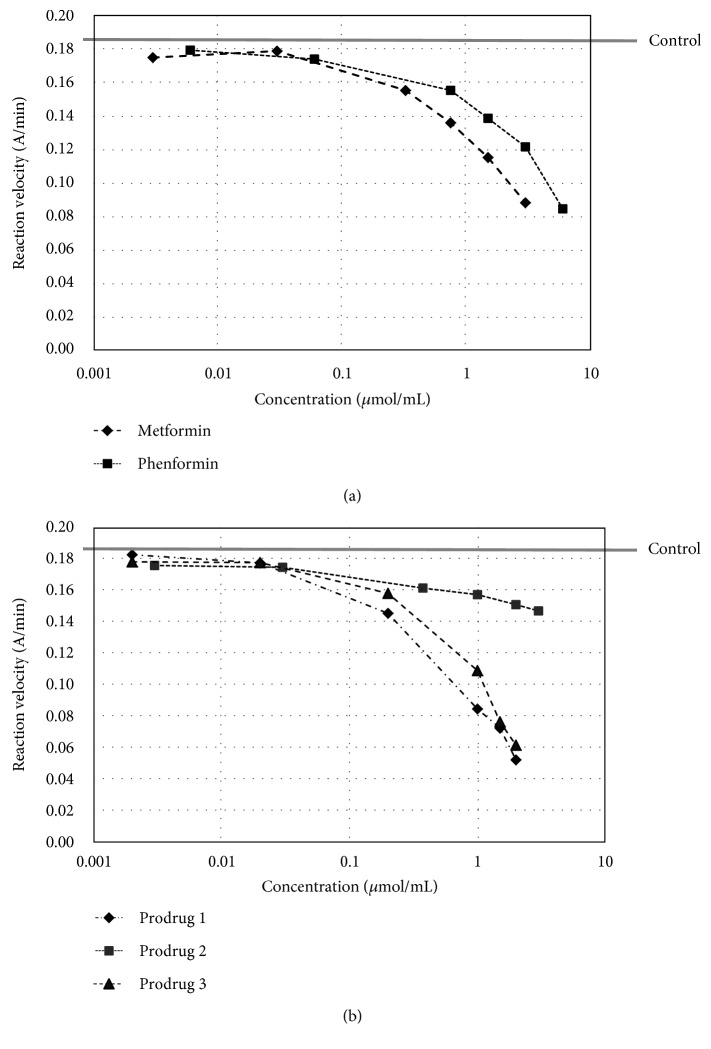
The effects of metformin and phenformin (a) and prodrugs 1, 2, and 3 (b) on AChE reaction velocity.

**Figure 3:**
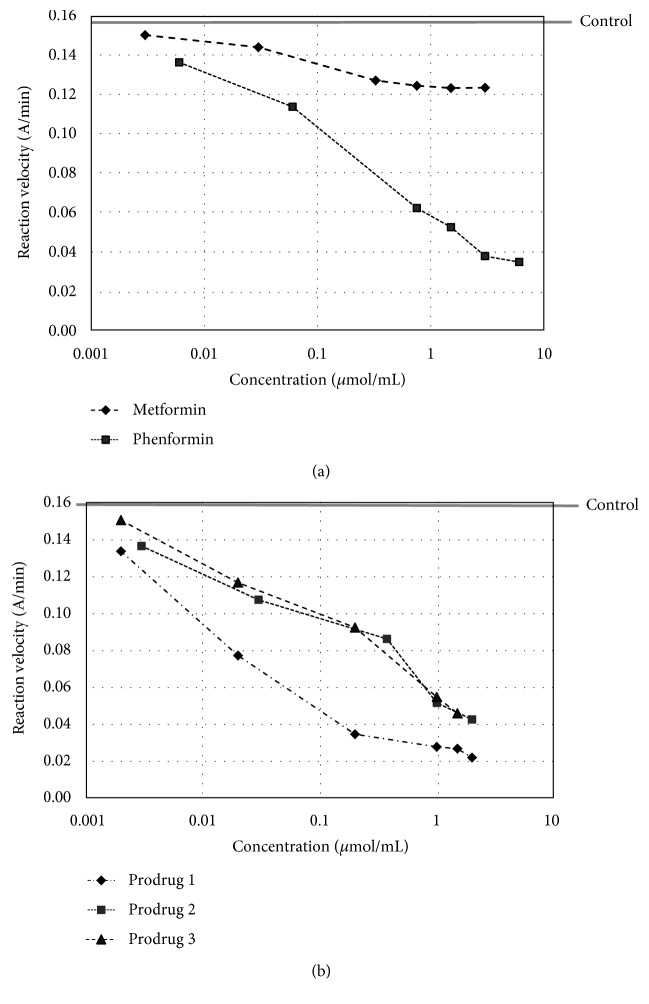
The effects of metformin and phenformin (a) and prodrugs 1, 2, and 3 (b) on BuChE reaction velocity. The velocity of pure BuChE was 0.160 A/min (grey line). The results are presented as a mean of three independent experiments. Phenformin and prodrugs 1, 2, and 3 inhibited the reaction depending on their concentration.

As presented in Figures 2 and 3, all examined compounds inhibited the activity of AChE; however, prodrug 2 only inhibited up to 21.2% at a concentration of 3 *μ*mol/mL. Similarly, in the case of BuChE, it was found that all compounds possess inhibitory properties except for metformin, which presented only weak anti-BuChE activity. The percentages of AChE and BuChE inhibition and IC_50_ values were then calculated on the basis of the reaction velocity (Table 1).

Tacrine, the first compound recommended by the FDA for the treatment of AD, was used to compare the obtained results [41]. Of the tested compounds, prodrug 1 demonstrated the highest activity towards human AChE (IC_50_ = 0.89 ± 0.157 *μ*mol/mL); however, this activity is much lower than that of tacrine (2.77∗10^−4^ ± 1.11∗10^–4^ *μ*mol/mL). Prodrug 1 appeared to be the most active also against BuChE (IC_50_ = 0.028 ± 0.002 *μ*mol/mL), whereas metformin inhibited BuChE so weakly (up to 26.4% at concentration of 3 *μ*mol/mL) that it did not allow IC_50_ to be calculated. On the basis of the calculated selectivity index (SI, Table 1), it was concluded that all other examined compounds exhibited higher selectivity toward BuChE than AChE.

### 3.2. Kinetic Parameters

**Figure 4:**
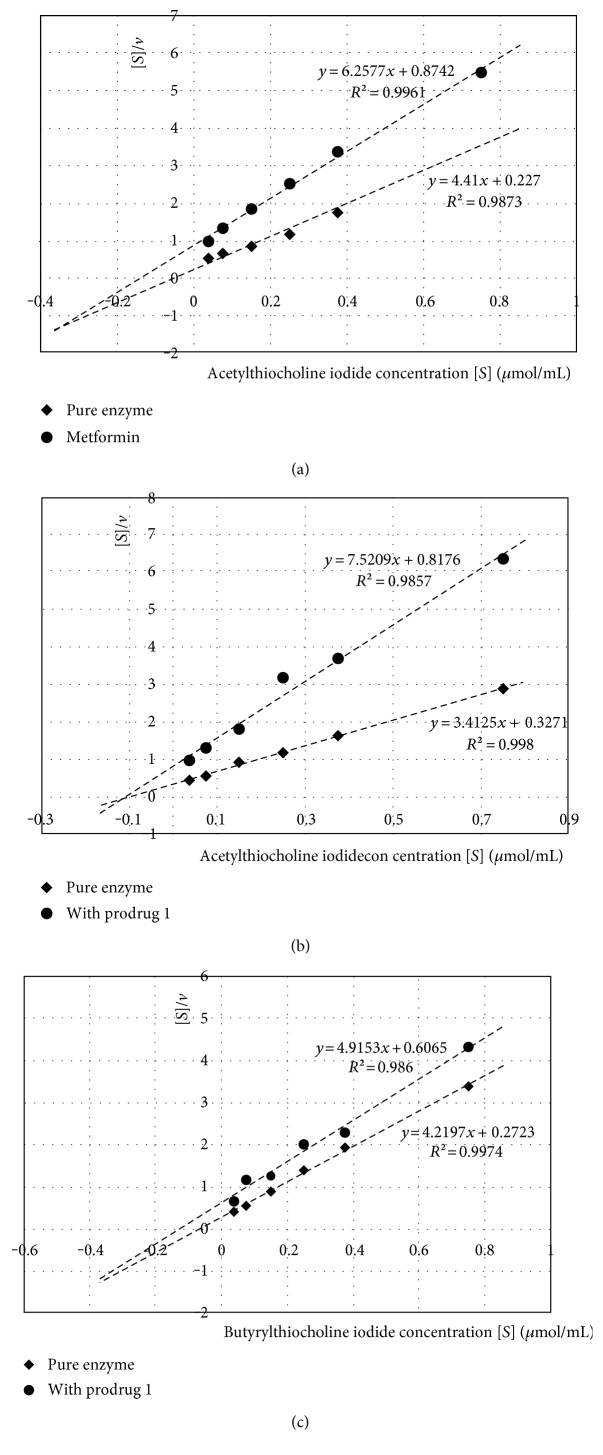
Hanes-Woolf's curves. (a) AChE and metformin at concentration of 2.35 *μ*mol/mL (IC_50_), mixed-type inhibition; (b) AChE and prodrug 1 at concentration of 0.89 *μ*mol/mL (IC_50_), noncompetitive inhibition; (c) BuChE and prodrug 1 at concentration of 0.028 *μ*mol/mL (IC_50_), mixed-type inhibition.

In order to determine the type of inhibition, additional experiments were conducted with various concentrations of substrates, and the kinetic parameters of the enzymatic reactions were obtained by linear regression using the Hanes-Woolf equations. The Hanes-Woolf (half-reciprocal) plot of [*S*]_0_/*v* against [*S*]_0_ gives intercepts at *K_m_*/*V*_max_ and *K_m_* (Figure 4).

The summarized results of *K_m_* and *V*_max_ are presented in Table 2. In Tables S1 and S2 available online at https://doi.org/10.1155/2017/7303096 (Supplementary materials), we included detailed data on each individual reaction (equations, *R*^2^, *K_m_*, and *V*_max_).

Comparing the *K_m_* and *V*_max_ values of the results obtained for pure enzyme and tested compounds allowed the type of inhibition to be determined (Figure 4). In the case of AChE inhibition, both metformin and phenformin exhibited mixed inhibition, as *V*_max(i)_ (*V*_max_ of the reaction with inhibitor) significantly decreased in comparison with *V*_max_ while *K*_*m*(i)_ (*K_m_* of the reaction with inhibitor) increased. Prodrugs 1 and 3 inhibited AChE noncompetitively (lack of changes between *K_m_* and *K*_*m*(i)_ and decreased *V*_max(i)_ value). Phenformin was shown to inhibit BuChE competitively, whereas noncompetitive inhibition was found for prodrug 3. In the case of prodrugs 1 and 2, the inhibition of BuChE was mixed type.

## 4. Discussion

A body of epidemiological data and pathophysiological evidence suggests the presence of various similarities between the two amyloidoses T2DM and AD. As noted in the Introduction, the two diseases present abnormal blood glucose levels, insulin resistance, inflammation, oxidative stress, and neurodegeneration [42, 43]. Nowadays, three ChE inhibitors can be used to delay the symptomatic decline observed in patients with AD. These drugs include AChE-selective inhibitors, such as donepezil and galantamine, and dual-acting AChE and BuChE inhibitor, such as rivastigmine. The first FDA-approved AChE-nonselective inhibitor, tacrine, is no longer routinely prescribed due to a high incidence of hepatotoxicity [44].

Due to its pleiotropic activity, metformin, the most frequently administered oral antidiabetic drug, has shown promise in the treatment of neurodegenerative diseases [28, 29]. For instance, Li et al. [33] determined AD-like brain changes in a mouse model of T2DM after treatment with metformin. The authors report that metformin administration for 18 weeks attenuated the increase of total tau, phospho-tau, and c-jun N-terminal kinase (JNK) activation. In addition, metformin weakened the reduction of synaptophysin, a synaptic protein, in mouse hippocampus. Furthermore, the results of this study imply that metformin did not attenuate the impairments of spatial learning and memory [33].

Despite its multidirectional pharmacological properties, metformin is characterized by unfavourable pharmacokinetics, as evidenced by slow and incomplete absorption from the intestine, resulting only in 50 to 60% bioavailability. In addition, intrasubject and intersubject variability has also been seen in its bioavailability, resulting in the response to metformin varying significantly, with approximately 30% of subjects receiving metformin being classified as nonresponders [45]. Therefore, there is a need to develop novel approaches, such as the synthesis of novel prodrugs of metformin in order to improve bioavailability [46].

No systematic study has yet examined the effects of metformin on ChEs. Therefore, the present study examines the ability of both recently synthesized prodrugs, clinically approved metformin and phenformin, which have been withdrawn from the market, to inhibit AChE and BuChE isolated from the human blood.

In spite of the results of Garcıa-Ayllon et al. [12] and their statement that plasma AChE might have potential as an indicator of AD progress and prognosis, we presume that the inhibitory activities of biguanides might be transferred into brain AChE. Nevertheless, we are aware of the different sensitivities of brain and RBC AChE and differences in their glycosylation (dimeric AChE from red cell membranes is more heavily glycosylated than the tetrameric brain enzyme) [47] which may affect the inhibitory properties of studied compounds. Plasma BuChE was used because soluble, globular tetrameric BuChE in plasma as well as the membrane-bound forms in the muscle and brain are encoded by the same BuChE mRNA. Serum BuChE was used in this study since its kinetic parameters were earlier found to be comparable to those obtained with BuChE isolated from the human brain tissue [48].

Both enzymes possess the capacity to hydrolyze ACh; however, they differ genetically, structurally, and kinetically [44]. Although BuChE represents only 10% of total ChE activity in a healthy human brain, it has been reported that the importance of BuChE in cholinergic neurotransmission is likely to increase in AD. This has been accounted for the presence of decreased AChE activity during the progression of AD [44]. Studies of rivastigmine use indicate that cognitive improvements correlate independently with the inhibition of AChE and BuChE in the cerebrospinal fluid of AD patients, which suggest that the inhibition of both esterases, a dual-acting property, is a highly desirable feature of AD therapy [49]. The importance of selective BuChE inhibition has further been shown using aged rats where BChE inhibition augmented ACh levels, increased cognitive function, and decreased amyloid deposits [50]. Since AChE activity decreases and BuChE activity increases as AD progresses, the inhibition of BuChE may become an increasingly important therapeutic target over time [43]. The principal objective of our studies was to evaluate in a systematic study the mechanism of AChE inhibition by metformin, as even the state-of-the-art scientific literature lacks such knowledge. Our goal was also to evaluate the effects of biguanides on BuChE activity, which now has emerged as important issue in AD treatment.

Our findings indicate that metformin inhibits 50% of AChE activity at a concentration of 2.35 ± 0.122 *μ*mol/mL and that it appears to be selective towards AChE, since it had very weak anti-BuChE activity (Table 1). This finding is supported by several studies. For instance, Bhutada et al. [51] have tested the influence of berberine, an isoquinolone alkaloid, against cognitive dysfunction in streptozotocin-induced diabetic rats. The authors assessed lipid peroxidation and glutathione levels as parameters of oxidative stress and ChE activity as a marker of cholinergic function. Induction of diabetes in rats contributed to a severe impairment in learning and memory associated with increased lipid peroxidation and ChE activity. Apart from berberine, the authors examined the influence of metformin and vitamin C. It was found that chronic treatment (30 days) with metformin at a dose of 500 mg/kg improved cognitive performance and reduced oxidative stress and ChE activity. No statistically significant effect on ChE activity was noted in the case of short-term administration of metformin (five days) [51]. Similarly, Saliua et al. [52] confirmed that metformin at a dose of 500 mg/kg significantly decreased AChE activity in the brain of streptozocin-induced diabetic rats [52]. Therefore, we may presume that the inhibitory effects of metformin on this key enzyme linked with neurodegeneration may be responsible for preventing cholinergic dysfunction in T2DM.

In contrast, some studies do not record any anti-ChE activity for metformin. For example, Arafa et al. [53] examined the effect of the antidiabetic medications canagliflozin and metformin on the levels of cortical neurotransmitters and ChE activity in a diabetic rat model. The authors report that the diabetic group exhibited a significant increase in AChE activity and a decrease in monoamine and amino acid neurotransmitter levels. A two-week treatment with canagliflozin led to decreased AChE activity, whereas the same treatment with metformin did not demonstrate significant influence on the enzyme activity [53]. Another study by Mostafa et al. found the application of metformin at doses of 100 and 300 mg/kg to have no effect on reduction of tissue AChE activity in a scopolamine-induced memory deficit rat model [36]. However, in both of these studies, the dose of metformin administered to animals was lower (100–300 mg/kg) than in those which report an influence on AChE activity (500 mg/kg).

In the present study, phenformin exhibited the lowest level of inhibition towards both ChE forms (the highest IC_50_ values in Table 1). Of the tested prodrugs, the sulfenamide with a cyclohexyl tail (prodrug 1) appeared to be the most active inhibitor for both AChE and BuChE (IC_50_ = 890 and 28 nmol/mL, resp.) thus demonstrating a dual-binding property that favours the inhibition of BuChE. Furthermore, these values are over 3000-fold higher for AChE, and 300-fold higher for BuChE, compared to those of tacrine, an AChE-nonselective inhibitor used clinically until 2013. With this in mind, it should be mentioned that compounds of natural origin with potential application as anti-AD agents are also much less potent than tacrine [39].

The sulfenamide prodrug with an octyl tail (prodrug 2) was the only compound which inhibited BuChE at nanomolar concentrations (184 nmol/mL, Table 1) but had very weak anti-AChE activity (IC_50_> 1000.0 *μ*mol/mL). Therefore, this prodrug can be regarded as a BuChE-selective inhibitor. BuChE selectivity appears to be important not only in AD but also in regard to inflammation, oxidative stress, and lipid metabolism [54]. For instance, it has been shown that streptozotocin-induced diabetic animals had dyslipidemia, increased plasma lipid peroxide content, decreased circulating plasma superoxide dismutase activity, and increased BuChE level [55]. In addition, elevated BuChE activity can lead to decreased ACh levels, thereby resulting in low-grade systemic inflammation [55]. Furthermore, there are also some studies that suggest that selective, reversible inhibition of brain BuChE may serve as a treatment for AD, improving cognition and modulating neuropathological AD markers such as inflammation [56].

Curiously, the sulfenamide prodrug with a shorter butyl tail (prodrug 3) was not selective towards BuChE and it had highest IC_50_ value towards both enzymes (1190 nmol/mL and 205 nmol/mL, resp., Table 1). Taken together, these results indicate that attaching a long alkyl or bulkier cyclohexyl chain to the opposite part of dimethyl group in metformin may improve its inhibition and selectivity towards BuChE.

Taking into consideration the type of inhibition by which prodrugs 1 and 3 inhibited AChE (noncompetitive, Table 2), our results illustrate that the prodrug molecules are able to bind to a site other than the catalytic active site (CAS) of the enzyme. It has been recognized that AChE has a peripheral anionic site (PAS) located at the aromatic-lined entrance of a narrow groove, on the bottom of which the CAS is located [57–59]. Therefore, it is likely that sulfenamide prodrugs of metformin can bind only to the PAS, which changes the enzyme's three-dimensional structure so that the CAS can still bind to substrates with the usual affinity; however, this is no longer the optimal arrangement for stabilizing the transition state and catalyzing the reaction. The ability to bind to the PAS arises most probably from the same structural properties (long or bulky side chain) that drive these prodrugs from AChE towards BuChE, since metformin and phenformin inhibited AChE in a mixed-type manner (Table 2), and while the smaller compound, metformin, inhibited BuChE only weakly (Table 1), phenformin was relatively more BuChE-selective. Phenformin was also the only compound which competitively inhibited BuChE (Table 2), which means that it was the only one that was able to compete with the substrate for the CAS of this enzyme. The bulkier sulfenamide prodrugs 1 and 2 inhibited BuChE with mixed-type manner and the smallest sulfenamide prodrug 3 did so purely noncompetitively. This is consistent with other studies that have claimed that the PAS is smaller in BuChE than in AChE [57].

When considering the properties of the prodrugs, the octylsulfenyl prodrug (2) is the only compound that has been shown to be stable both in vitro and in vivo [38], while both cyclohexyl and butyl sulfenyl prodrugs (1 and 3) have shown to be bioconverted quickly not only in vitro in the presence of free thiols but also in vivo in cells such as erythrocytes which are rich in endogenous thiol [37, 38, 60]. Therefore, prodrug 1 and 3 are less likely to be delivered intact across the blood-brain barrier (BBB), while prodrug 2 is more likely to reach the extracellular fluids (ECF) and the synaptic clefts within the brain, where the ChEs are located. However, even though prodrugs 1 and 3 are bioconverted to metformin soon after their oral absorption [37, 38], they can improve the oral bioavailability of metformin; according to these results, they can therefore be considered as prodrugs of a selective AChE inhibitor (metformin).

Taken together, the results gained in this study offer encouragement in the development of a new class of selective and unselective ChE inhibitors with a biguanide backbone structure. If a prodrug property is not desired, the linking sulphur atom can also be left out to stabilize the structure. Increasing the chain length and size, it is possible to achieve BuChE-selective inhibitors, while the presence of small side chains on both sides of the biguanides ensures the dual binding property. The use of selective ChE inhibitors would allow more detailed study of the function and role of AChE and BuChE in AD in the future. On the other hand, the capacity for simultaneous interaction with PAS and resulting dual-binding potential to both ChEs is an attractive property in the rational design of ChE inhibitors, since binding to PAS has also been associated with an ability to interfere with amyloid-*β* deposition and aggregation [61].

## 5. Summary

The aim of the present in vitro study was to determine the inhibitory properties of metformin and its sulfenamide prodrugs towards ChEs. Metformin was found to moderately inhibit the activity of AChE in a mixed-type inhibition and to have very weak anti-BuChE activity. These results may contribute to a better understanding of the neuroprotective role of the most frequently used antidiabetic drug, metformin. Conversely, a sulfenamide prodrug containing an eight-carbon alkyl chain presented weak activity towards AChE inhibition, but nanomolar inhibition towards BuChE. Two other sulfenamide prodrugs inhibited AChE and BuChE noncompetitively or with a mixed-type pattern. Therefore, these results together indicate that the bulky side chains of biguanides are most likely to interact with the PAS of AChE and drive the compounds towards BuChE-selective inhibition, while drugs with smaller side chains are more likely to retain the noncompetitive inhibitory activity for both enzymes and thus have dual-binding properties. Thus, biguanides might have potential in preventing brain disorders associated with diabetes complications in future.

## Supplementary Material

Table S1. Equations, R2, and kinetic parameters of AChE reactions. Table S2. Equations, R2, and kinetic parameters of BuChE reactions.

## Figures and Tables

**Table 1:** Effects of metformin, phenformin, and metformin prodrugs on the human erythrocyte acetylcholinesterase (AChE) and plasma butyrylcholinesterase (BuChE).

Compound	IC_50_ [*μ*mol/mL]		SI	
	AChE	BuChE	AChE	BuChE
Metformin	2.350 ± 0.122	>1000.000^×^	>425.531^×^	<0.002^×^
Phenformin	4.940 ± 0.575	0.259 ± 0.031	0.052	19.073
Prodrug 1	0.890 ± 0.157	0.028 ± 0.002	0.031	31.786
Prodrug 2	>1000.000^×^	0.184 ± 0.014	<1.840∗10^−4^^×^	>5434.000^×^
Prodrug 3	1.190 ± 0.139	0.205 ± 0.029	0.172	5.805
*Tacrine* ^∗^	(2.770 ± 1.11)∗10^−4^	(9.080 ± 4.54)∗10^−5^	3.278	0.305

The values are given as mean ± standard deviation (SD) in three independent experiments. Values of IC_50_ for tacrine^∗^ were published previously [41]. The above values for tacrine were calculated from units *μ*g/mL (0.055 ± 0.022 and 0.018 ± 0.009, resp.). SI (selectivity index)—the AChE selectivity index is defined as IC_50_ BChE/IC_50_ AChE affinity ratio. Selectivity for BChE is defined as IC_50_(AChE)/IC_50_(BChE). ^×^Theoretical values counted on the basis of extrapolated plots for metformin towards BuChE and prodrug 2 towards AChE. SI was calculated based on the theoretical IC_50_ values.

**Table 2:** Kinetic parameters of enzymatic reactions.

Compound		AChE			BuChE		
		*K_m_* [*μ*mol/mL]	*V*_max_ [A/min]	I	*K_m_* [*μ*mol/mL]	*V*_max_ [A/min]	I
Metformin	A	0.056 ± 0.012	0.224 ± 0.002	M	NS	NS	—
	B	0.167 ± 0.024	0.164 ± 0.004		NS	NS	

Phenformin	A	0.056 ± 0.028	0.230 ± 0.017	M	0.037 ± 0.015	0.159 ± 0.045	C
	B	0.083 ± 0.047	0.127 ± 0.003		0.152 ± 0.029	0.167 ± 0.039	

Prodrug 1	A	0.091 ± 0.004	0.292 ± 0.008	NC	0.044 ± 0.025	0.222 ± 0.047	M
	B	0.097 ± 0.018	0.132 ± 0.003		0.087 ± 0.047	0.194 ± 0.008	

Prodrug 2	A	NS	NS	—	0.046 ± 0.020	0.202 ± 0.019	M
	B	NS	NS		0.078 ± 0.024	0.091 ± 0.015	

Prodrug 3	A	0.089 ± 0.003	0.290 ± 0.002	NC	0.045 ± 0.021	0.202 ± 0.019	NC
	B	0.078 ± 0.012	0.159 ± 0.026		0.054 ± 0.021	0.117 ± 0.022	

The values are given as mean ± standard deviation (SD) in 3 independent experiments. NS—the kinetic parameters were not estimated. A—kinetic parameters for pure enzyme (*K_m_*, *V*_max_); B—kinetic parameters of tested compounds (inhibitors) (IC_50_ concentrations) (*K*_*m*(i)_, *V*_max(i)_). I—type of inhibition, M—mixed type, NC—inhibition noncompetitive, C—inhibition competitive.
