# Supplementary material for: Metformin and Its Sulfenamide Prodrugs Inhibit Human Cholinesterase Activity
Source: Oxid Med Cell Longev. 2017 Jul 9;2017:7303096. doi: 10.1155/2017/7303096 (PMC5523189; doi:10.1155/2017/7303096)
Supplement: Supplementary file 1 — Table S1. Equations, R2, and kinetic parameters of AChE reactions. Table S2. Equations, R2, and kinetic parameters of BuChE reactions. [file 7303096.f1.docx]

***Table S1.*** *Equations, R^2^, and kinetic parameters of AChE reactions.*

| **COMPOUND** | | **AChE** | | | | | | |
| --- | --- | --- | --- | --- | --- | --- | --- | --- |
|  |  | **Equation** | **R^2^** | **Km [µmol/mL]** | **Vmax [A/min]** | **Mean ± SD** | | |
|  |  |  |  |  |  | **A, B** | **Km [µmol/mL]** | **Vmax [A/min]** |
| Metformin | A_1_ | y = 4.3933x + 0.306 | 0.9905 | 0.069 | 0.225 | A | 0.056 ±0.012 | 0.224 ± 0.002 |
|  | B_1_ | y = 5.9545x + 1.1126 | 0.9906 | 0.186 | 0.167 |  |  |  |
|  | A_2_ | y = 4.4886x + 0.2113 | 0.9824 | 0.047 | 0.222 |  |  |  |
|  | B_2_ | y = 6.08x + 1.0619 | 0.9909 | 0.175 | 0.165 | B | 0.167 ± 0.024 | 0.164 ± 0.004 |
|  | A_3_ | y = 4.41x + 0.227 | 0.9873 | 0.051 | 0.226 |  |  |  |
|  | B_3_ | y = 6.2577x + 0.8742 | 0.9961 | 0.139 | 0.159 |  |  |  |
| **Phenformin** | A_1_ | y = 4.0139x + 0.3354 | 0.9948 | 0.084 | 0.250 | A | 0.056 ± 0.028 | 0.230 ± 0.017 |
|  | B_1_ | y = 7.9356x + 0.4511 | 0.9590 | 0.057 | 0.126 |  |  |  |
|  | A_2_ | y = 4.5274x + 0.1278 | 0.9886 | 0.028 | 0.219 |  |  |  |
|  | B_2_ | y = 7.7411x + 1.0576 | 0.9842 | 0.137 | 0.130 | B | 0.083 ± 0.047 | 0.127 ± 0.003 |
|  | A_3_ | y = 4.5217x + 0.2585 | 0.9942 | 0.057 | 0.221 |  |  |  |
|  | B_3_ | y = 7.9589x + 0.4375 | 0.9749 | 0.055 | 0.125 |  |  |  |
| **Prodrug 1** | A_1_ | y = 3.3339x + 0.2963 | 0.9979 | 0.088 | 0.299 | A | 0.091 ± 0.004 | 0.292 ± 0.008 |
|  | B_1_ | y = 7.7811x + 0.5958 | 0.9747 | 0.076 | 0.128 |  |  |  |
|  | A_2_ | y = 3.4125x + 0.3271 | 0.9980 | 0.095 | 0.293 |  |  |  |
|  | B_2_ | y = 7.5209x + 0.8176 | 0.9857 | 0.108 | 0.133 | B | 0.097 ± 0.018 | 0.132 ± 0.003 |
|  | A_3_ | y = 3.5297x + 0.3097 | 0.9990 | 0.087 | 0.283 |  |  |  |
|  | B_3_ | y = 7.4639x + 0.8002 | 0.9925 | 0.107 | 0.133 |  |  |  |
| **Prodrug 3** | A_1_ | y = 3.4655x + 0.3171 | 0.9974 | 0.091 | 0.288 | A | 0.089 ± 0.003 | 0.290 ± 0.002 |
|  | B_1_ | y = 7.3382x + 0.4859 | 0.9948 | 0.066 | 0.136 |  |  |  |
|  | A_2_ | y = 3.4273x + 0.295 | 0.9960 | 0.086 | 0.291 |  |  |  |
|  | B_2_ | y = 5.3301x + 0.4858 | 0.9974 | 0.091 | 0.187 | B | 0.078 ± 0.012 | 0.159 ± 0.026 |
|  | A_3_ | y = 3.4373x + 0.3044 | 0.9955 | 0.088 | 0.290 |  |  |  |
|  | B_3_ | y = 6.5036x + 0.5063 | 0.9949 | 0.077 | 0.153 |  |  |  |

A – kinetic parameters for pure enzyme (K_m_, v_max_); B – kinetic parameters of tested compounds (inhibitors) (IC_50_ concentrations) (K_m(i)_, v_max(i)_); numerical index A_1_, A_2_ – the number of individual experiment (all experiments were conducted three times on different biological samples).

***Table S2.*** *Equations, R^2^, and kinetic parameters of BuChE reactions.*

| **COMPOUND** | | **BuChE** | | | | | | |
| --- | --- | --- | --- | --- | --- | --- | --- | --- |
|  |  | **Equation** | **R^2^** | **Km [µmol/mL]** | **Vmax [A/min]** | **Mean ± SD** | | |
|  |  |  |  |  |  | **A, B** | **Km [µmol/mL]** | **Vmax [A/min]** |
| **Phenformin** | A_1_ | y = 8.4335x + 0.1978 | 0.9991 | 0.023 | 0.116 | A | 0.037 ± 0.015 | 0.159 ± 0.045 |
|  | B_1_ | y = 7.9168x + 1.0419 | 0.9736 | 0.132 | 0.127 |  |  |  |
|  | A_2_ | y = 4.891x + 0,1707 | 0.9989 | 0.035 | 0.205 |  |  |  |
|  | B_2_ | y = 4.8826x + 0.6743 | 0.9956 | 0.138 | 0.205 | B | 0.152 ± 0.029 | 0.167 ± 0.039 |
|  | A_3_ | y = 6.4402x + 0.3404 | 0.9865 | 0.053 | 0.156 |  |  |  |
|  | B_3_ | y = 5.8875x + 1.0906 | 0.9538 | 0.185 | 0.170 |  |  |  |
| **Prodrug 1** | A_1_ | y = 4.7479x + 0.2442 | 0.9987 | 0.051 | 0.210 | A | 0.044 ± 0.025 | 0.222 ± 0.014 |
|  | B_1_ | y = 5.2967x + 0.5488 | 0.9849 | 0.103 | 0.188 |  |  |  |
|  | A_2_ | y = 4.2197x + 0.2773 | 0.9974 | 0.064 | 0.236 |  |  |  |
|  | B_2_ | y = 4.9153x + 0.6065 | 0.9860 | 0.123 | 0.203 | B | 0.087 ± 0.047 | 0.194 ± 0.008 |
|  | A_3_ | y = 4.5915x + 0.0772 | 0.9952 | 0.016 | 0.217 |  |  |  |
|  | B_3_ | y = 5.2989x + 0.1831 | 0.9974 | 0.034 | 0.188 |  |  |  |
| **Prodrug 2** | A_1_ | y = 4.7479x + 0.2442 | 0.9987 | 0.051 | 0.210 | A | 0.046 ± 0.020 | 0.202 ± 0.019 |
|  | B_1_ | y = 8.7899x + 0.8634 | 0.9866 | 0.098 | 0.099 |  |  |  |
|  | A_2_ | y = 4.6401x + 0.2922 | 0.9992 | 0.062 | 0.215 |  |  |  |
|  | B_2_ | y = 13.666x + 1.1438 | 0.9746 | 0.083 | 0.073 | B | 0.078 ± 0.024 | 0.091 ± 0.015 |
|  | A_3_ | y = 5.5314x + 0.1347 | 0.9975 | 0.024 | 0.180 |  |  |  |
|  | B_3_ | y = 9.9847x + 0.5183 | 0.9838 | 0.051 | 0.100 |  |  |  |
| **Prodrug 3** | A_1_ | y = 4.7479x + 0.2442 | 0.9987 | 0.051 | 0.210 | A | 0.045 ± 0.021 | 0.202 ± 0.019 |
|  | B_1_ | y = 7.2351x + 0.3786 | 0.9953 | 0.052 | 0.138 |  |  |  |
|  | A_2_ | y = 4.6401x + 0.2922 | 0.9992 | 0.062 | 0.215 |  |  |  |
|  | B_2_ | y = 8.5157x + 0.6537 | 0.9864 | 0.076 | 0.117 | B | 0.054 ± 0.021 | 0.117 ± 0.022 |
|  | A_3_ | y = 5.5513x + 0.1205 | 0.9958 | 0.021 | 0.180 |  |  |  |
|  | B_3_ | y = 10.55x + 0.3625 | 0.9909 | 0.034 | 0.094 |  |  |  |

A – kinetic parameters for pure enzyme (K_m_, v_max_); B – kinetic parameters of tested compounds (inhibitors) (IC_50_ concentrations) (K_m(i)_, v_max(i)_); numerical index A_1_, A_2_ – the number of individual experiment (all experiments were conducted three times on different biological samples).
